# Etiological and Epidemiological Characteristics of Severe Mastitis and the Outcomes Treatment Following a Single Dose of Fluoroquinolones Administered During On-Farm Veterinary Interventions

**DOI:** 10.3390/antibiotics15060538

**Published:** 2026-05-25

**Authors:** Olivier Salat, Philippe Pottié, Nolwenn Prigent, Catherine Lutz, Alicia Nurit, Vincent Herry, Arnaud Sartelet, Charly De Campos, Laurent Dravigney

**Affiliations:** 1 Veterinary Clinic of Haute Auvergne, 15100 Saint Flour, France; l.dravigney@gmail.com; 2 Veterinary Clinic of Tétras Lyre, 74520 Valleiry, France; pottie.gtv74@gmail.com; 3 Veterinary Clinic of the Souleuvre, 14350 Souleuvre en Bocage, France; nol.prigent@orange.fr; 4 Rumi Passion, 67270 Hochfelden, France; cl@rumipassion.fr; 5 Optivet, 01190 Saint Bénigne, France; alicia.nurit@optivet.fr; 6 Veterinary Clinic EVA, 79150 Argentonnay, France; vincentherry86@gmail.com; 7 Veterinarians South Mayenne, 53400 Craon, France; a.sartelet@vetsudmayenne.fr; 8 Department of Livestock Diseases, National Veterinary School of Toulouse, 31300 Toulouse, France; charly.decampos@envt.fr

**Keywords:** severe mastitis, treatment, fluoroquinolones, field study, etiology, epidemiology

## Abstract

Background/objectives: severe mastitis is one of the leading causes of mortality in dairy cows. Its primary complication is shock, predominantly associated with systemic inflammatory response syndrome, which remains extremely challenging for practitioners to manage. The average mortality rate is estimated at approximately 25%. Many authors recommend the use of fluoroquinolones for this indication. However, these antibiotics are classified as critically important for human health, and their use requires strict compliance with specific guidelines (bacteriological analysis and antimicrobial susceptibility testing). In addition, some practitioners remain reluctant to use this class of antibiotics in field conditions. Therefore, the present study aimed to evaluate the outcomes of systematic antibiotic therapy using fluoroquinolones in cases of severe mastitis and to identify factors that may influence treatment success. Methods: a total of 323 cows with severe mastitis were enrolled by eight participating veterinary clinics located across different regions of France. The study design included: (i) clinical scoring based on a standardized grid developed by practitioners routinely managing this condition, (ii) bacteriological analysis of milk samples (with antimicrobial susceptibility testing performed when Gram-negative bacteria were isolated), and (iii) post-treatment follow-up consisting of telephone interviews conducted at 5 and 15 days after inclusion. Cows presenting with a clinical score ≥3 (scale 0–36) in association with local signs of mastitis were classified as having severe mastitis and received an injection of 10 mg/kg marbofloxacin along with 2.2 mg/kg flunixin (unless another NSAID had been administered within the previous 24 h). When the clinical score was ≥6, cows additionally received intravenous fluid therapy consisting of 3 L of 7.2% NaCl, supplemented by oral drenching if spontaneous water intake was insufficient. Results: a total of 43 cows died or were euthanized during the study period, corresponding to a mortality rate of 13.3%. The mean clinical score at inclusion was 12.6. The clinical signs most strongly associated with mortality were decubitus and hypothermia at admission. *Escherichia coli* was isolated in 67.0% of severe mastitis cases, either as a single pathogen (82.9%) or in mixed infections (17.1%). Overall, Gram-negative bacteria (*Escherichia coli*, *Klebsiella* spp., *Pseudomonas aeruginosa*, other Gram-negative organisms) were identified in 79.0% of cases. A total of 188 coliform isolates were tested for antimicrobial susceptibility. All isolates (100%) were susceptible to marbofloxacin, as were all tested Gram-negative strains, whereas only 79.9% of *E. coli* isolates were susceptible to sulfonamide/trimethoprim. Compared with previously published data, the observed mortality rate was lower despite the poor clinical condition of cows at admission. Conclusion: the timeliness of initiating effective antimicrobial therapy appears to be a critical determinant of survival in cows with severe mastitis.

## 1. Introduction

Severe mastitis represents the leading cause of intervention by cattle practitioners in the context of udder health. These conditions are life-threatening and result in substantial economic losses. They are often considered the primary cause of mortality in dairy herds [1,2]. Reported mortality rates range from 13.5 and 35.0%, with overall economic losses amounting to several billion euros. Multiple mechanisms contribute to the pathophysiology of severe mastitis, including intense local inflammation leading to the release of large quantities of inflammatory mediators [3], frequent endotoxemia [4,5] and, in the most severe cases, septicemia [5,6,7]. The use of antibiotics in the treatment of severe mastitis, particularly cases caused by *E. coli*, has long been debated [8,9]. However, parenteral antimicrobial therapy is now widely considered essential [7,10,11,12].

Severe mastitis cases observed under field conditions are difficult to reproduce through experimental inoculation. The severity of these cases is thought to be primarily determined by the rapidity of the host immune response within the udder following infection [3]. Consequently, early and effective treatment is likely to be critical in limiting disease progression and improving survival outcomes. The objectives of this observational study were, first, to provide a detailed description of the disease, including its etiological agents, the categories of cows most affected and the stages of lactation at higher risk, and second, to accurately assess its clinical severity using a standardized scoring system. An additional objective was to evaluate the clinical and zootechnical outcomes of a systematic treatment protocol combining a fluoroquinolone with a non-steroidal anti-inflammatory drug (NSAID), with adjunctive fluid therapy administered when indicated.

## 2. Results

A total of 324 cases of severe mastitis were initially enrolled in the study. However, only 323 cases were retained for the final analysis, as one case presented with a clinical score <3. Complete data were not available for all 323 cases; however, the majority of records were sufficiently complete for analysis. Consequently, the total number of observations varies depending on the parameter evaluated. The cases were recruited from 185 farms, of which 128 contributed a single case and 57 contributed multiple cases. Two farms contributed up to 12 cases each. At the time of inclusion, 83 cows (25.7%) were already receiving antimicrobial treatment. These treatments primarily consisted of penethamate, injectable combinations of sulfonamides and trimethoprim, penicillin G, or intramammary formulations combining neomycin, bacitracin and tetracycline.

### 2.1. Epidemiology of Severe Mastitis

Severe mastitis predominantly affects older cows. Cows in their third lactation or higher account for approximatively three-quarters of cases, despite typically representing less than one-third of the dairy herd (see Table 1). More than one-quarter of cases occurred during the peripartum period, whereas the majority (68.2%) developed during the first half of lactation (Table 2). Severe mastitis occurred in approximatively one out of six cases around calving, either immediately before or after parturition.

### 2.2. Clinical Signs

The prevalence of the main clinical signs affecting general condition is summarized in Table 3 (see Table S1). At the time of inclusion, eight cows presented with gangrene and eight with diarrhea. Clinical scores ranged from 3 to 31, with a mean value of 12.6. A total of 92.3% of cows had a clinical score ≥6 and therefore received fluid therapy. At inclusion, 26.3% of cows were recumbent.

### 2.3. Bacteriological Analyses

Bacteriological analyses performed in veterinary laboratories resulted in the isolation of a single bacterial species in 280 cases (Table 4), two species in 40 cases (Table 5) and more than two species in 3 cases. These latter samples were therefore considered contaminated. These results highlight the marked predominance of Enterobacteriaceae, with *E. coli* being by far the most frequently isolated organism in the milk samples. The combination of *E. coli* and *S. uberis* was the most common in cases involving dual bacterial isolation. Among the eight cows presenting with gangrenous mastitis, four yielded an ‘other Gram-positive’ bacteriological result, two had coliform bacteria isolated from the udder, one had a mixed infection with *E. coli* and *S. uberis*, and one sample was classified as ‘contaminated’.

Only antimicrobial susceptibility results for coliform bacteria are reported (see Table 6), as the number of isolates was sufficient to ensure representative findings.

### 2.4. Health and Economic Consequences

A total of 43 cows died or were euthanized within 15 days following inclusion for severe mastitis. This corresponding to a mortality rate of 13.3%.

**Figure 1:**
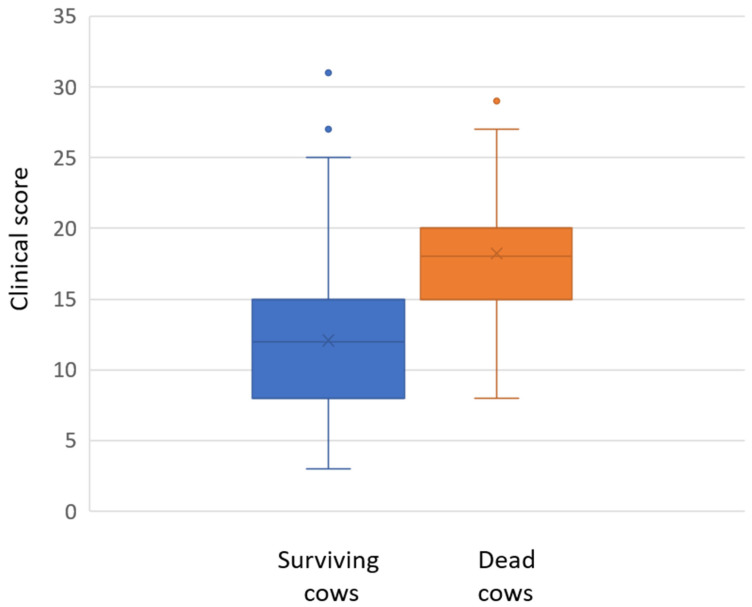
Comparison of the distribution of clinical scores between survivors and non-survivors.

**Figure 2:**
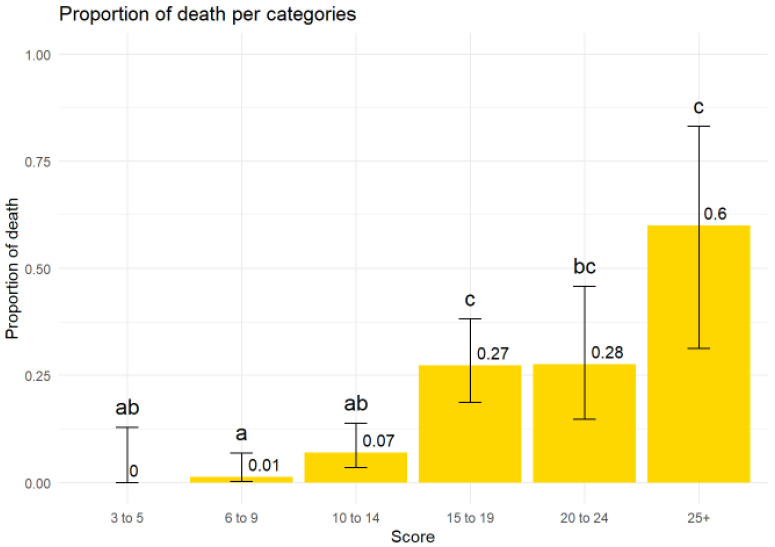
Comparison of mortality rates according to clinical score at the inclusion (a ≠ b: *p* < 0.05; a ≠ c; *p* < 0.05, b ≠ c: *p* < 0.05).

Complete clinical records were available for 320 cows (3 missing datasets), including 277 survivors and 43 cows that died or were euthanized. The mean clinical score in cows that died or were euthanized was 18.2, compared with 12.1 (*p* < 0.01) in cows that were alive and clinically healthy at 15 days post-inclusion (Table 7, Figure 1). The mortality rate increased with the clinical score at inclusion (see Table 8, Figure 2). Three of the eight cows presenting with gangrenous mastitis did not survive.

**Figure 3:**
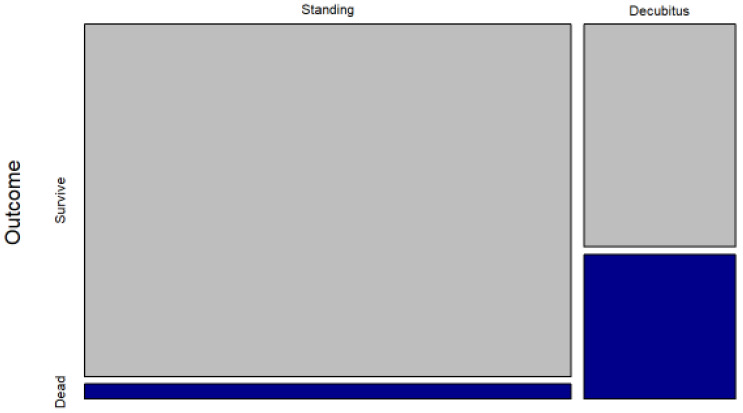
Comparison of the proportion of deaths among standing and recumbent cows at the start of the study (*p* < 0.0001).

**Figure 4:**
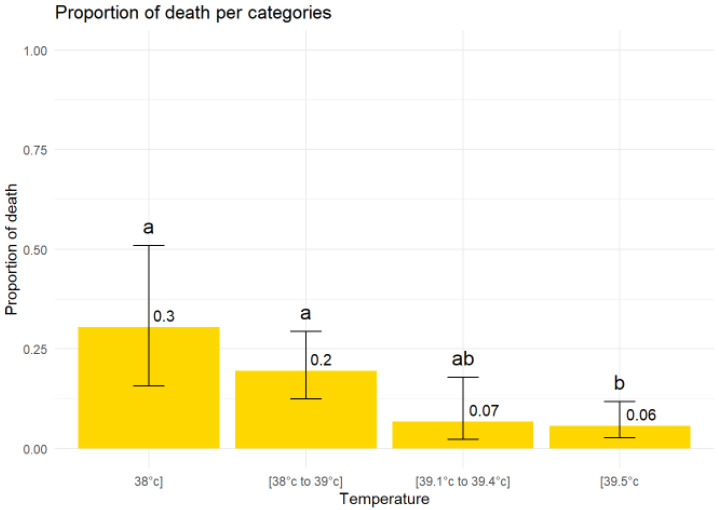
Comparison of mortality rates according to body temperature at admission (a ≠ b: *p* < 0.001). (38 °c] = <38.0 °c; [39.5 °c = >39.5 °c).

The clinical parameter most strongly associated with mortality was recumbency. Nearly 40% of cows that were recumbent at the time of inclusion died or were euthanized, whereas only 4.1% of cows that were standing at inclusion did not complete the study (*p* < 0.0001, Figure 3). The lowest mortality rate (5.6%) was observed in cows with hyperthermia at inclusion, compared with normothermic cows (19.5%) or hypothermic cows (30.4%) (Figure 4).

The pathogens isolated in association with these outcomes varied, although *E. coli* predominated (see Table 9).

Fifteen days after the clinical episode, the impact of severe mastitis on milk production (Table 10) and on the affected quarter (Table 11) were assessed.

## 3. Discussion

This study is based on an evaluation of the clinical consequences of severe mastitis and the outcomes of initial fluoroquinolone treatment. The success of antimicrobial therapy for intramammary infections is primarily assessed by bacteriological cure. This is generally the case for most forms of mastitis, except in severe cases, where the inflammatory response is particularly intense. In such cases, exacerbation of this inflammation represents a greater clinical challenge than pathogen elimination. Accordingly, most studies focusing on severe mastitis report clinical recovery as the primary endpoint [5,7,13,14]. Clinical and zootechnical outcomes were assessed via structured telephone interviews conducted 5 days and 15 days after inclusion. Although this method may introduce bias, data collection was strictly standardized, with predefined response categories. Quantitative measurements would have been more objective but were not feasible in a field setting. In particular, farmers were asked to estimate whether milk yield was above or below 50% of pre-mastitis production, which represents a potential source of classification bias; however, any misclassification is likely to be nondifferential.

The classification of severe mastitis used in this study is based on Wenz et al. [15], focusing on systemic clinical signs rather than local udder abnormalities. This approach is justified, as systemic signs are not consistently correlated with local lesions [3], particularly in the peripartum period, where diagnostic delays may occur if there are no obvious local signs. The clinical score was therefore constructed using systemic parameters (rectal temperature, enophthalmos, depression, ruminal motility), with additional indicators including ruminal fill, locomotion, ability to stand, skin tent duration as an additional indicator of dehydration, and scleral injection as a marker associated with septicemia [16].

The clinical signs observed and their frequency at inclusion were consistent with previous reports [17,18]. Overall, the clinical scores confirm the severity of the condition. The threshold for initiating fluid therapy (score ≥6) was supported by outcome data, as the lowest score among non-surviving cows was 8. However, clinical evolution over time could not be monitored due to logistical constraints. Cows were considered clinically recovered when they were standing and voluntarily eating, as assessed during follow-up calls.

Severe mastitis was more frequent in older cows, consistent with previous studies showing increased risk with parity [3,19,20,21]. Although a substantial proportion of cases occurred during the peripartum period (>25%), cases were distributed throughout lactation, with many occurring in mid- to late lactation. Approximately 5% of cases occurred shortly after drying off, potentially reflecting suboptimal hygiene during intramammary dry-cow therapy.

Gram-negative bacteria, particularly *E. coli*, predominated (≈80% of isolates), a proportion higher than previously reported [2,3,4,5,6,7,8,9,10,11,12,13,14,15,16,17,18,19,20,21,22,23,24]. This likely reflects case severity, as severe clinical presentations are more frequently associated with Enterobacteriaceae such as *Escherichia coli* or *Klebsiella* spp. [17,25]. Culture-negative results were rare (3.6%), in contrast to other studies [12,22], likely due to higher bacterial loads and case severity. A potential limitation is the inability of the culture method used to detect anaerobic organisms.

This study is, to our knowledge, the first to report antimicrobial susceptibility data specifically for *Escherichia coli* isolated from severe mastitis cases. Susceptibility patterns were broadly consistent with previous reports in mastitis populations not stratified by severity [26,27], although a higher proportion of resistance to sulfonamide/trimethoprim was observed. No ESBL-producing *E. coli* were detected.

Severe mastitis is associated with a marked systemic inflammatory response and may progress to sepsis, explaining the high mortality rates reported (approximately 25%) [28], with substantial variability across studies (13.5–35% or higher) [5,6,7,13,14,28]. These differences likely reflect variation in case definition, pathogen distribution (particularly Gram-negative involvement), clinician involvement (veterinarians [29] vs. technicians [13,22]), and treatment protocols. The use of a standardized clinical score in this study allowed objective severity stratification and uniform initial treatment.

Hypertonic saline infusion (3 L) was systematically administered in cases with a clinical score ≥6, in line with recommendations for hypovolemic shock management [30]. This protocol was applied uniformly during the first 24 h. Subsequent antimicrobial adjustments were made based on bacteriological results, mainly targeting Gram-positive infections (penicillin G or tylosin). These additional treatments were not considered treatment failures, as early outcome within the first 24 h was considered the most relevant prognostic window.

Designing a randomized field trial with a negative control group would be ethically and practically challenging given disease severity. Comparisons with the literature are further limited by heterogeneity in inclusion criteria and therapeutic protocols. Only one study presents partially comparable conditions [29], although important differences exist regarding antimicrobial choice, absence of fluoroquinolones, and lack of standardized clinical scoring. Compared with this study, our population included more recumbent cows (26.3% vs. 15.0%), more frequent use of fluid therapy (92.3% vs. 81.4%), and less calcium supplementation (25.1% vs. 53.0%), while mortality was approximately twofold lower.

Interestingly, cows with hyperthermia at inclusion showed lower mortality. Given that hyperthermia is typically transient in severe mastitis (12–24 h) [25], early intervention during this phase may improve survival outcomes.

Beyond mortality, severe mastitis had major economic consequences, with approximately 40% of cows experiencing a marked reduction in milk yield and two-thirds ceasing production. These findings are consistent with previous reports [28], which also describe high culling rates and quarter loss following severe mastitis episodes.

Finally, early fluid therapy guided by clinical scoring (≥6) appears justified and consistent with recommendations for sepsis management [31].

There is broad consensus that severe mastitis requires parenteral antimicrobial therapy [10,32]. Fluoroquinolones have demonstrated strong efficacy due to their pharmacokinetic properties and activity against major pathogens involved in severe mastitis [10,33]. Their use is particularly relevant in high bacterial load infections, where rapid reduction in pathogen burden is critical to limiting inflammation and preventing septicemia [3,5,23,34,35,36]. However, conflicting field studies exist [27,37], although both present important methodological limitations, including heterogeneity in severity classification, lack of randomization, and imbalance between treatment groups [38]. As such, their conclusions should be interpreted with caution. Overall, no robust evidence currently demonstrates the efficacy of non-critical antibiotics in severe mastitis, the only available study [39] also has significant biases, particularly regarding the severity of the conditions. While fluoroquinolones must be used judiciously due to concerns regarding antimicrobial resistance [40], this study provides field-based data on their use under real-world conditions. No fluoroquinolone-resistant Gram-negative isolates were detected. The absence of resistance emergence may be related to single-dose administration and restricted use to severe mastitis cases.

## 4. Material and Methods

Participating veterinary clinics: Eight veterinary practices participated in this study; all located in the main dairy regions of France. Each practice routinely performed on-site bacteriological analyses using a standardized culture protocol. As part of the study agreement, all clinics committed to systematically recording clinical parameters, applying a standardized treatment protocol, and ensuring follow-up of all included cases. A shared Excel database was used to record case-level data and monitor study progress in real time. The data were collected during farm visits conducted for the management of severe mastitis cases. For various reasons (oversights, farmer unavailability, delayed recording), some clinical records were incomplete. Whenever possible, they were completed retrospectively when the information was still available.

Case inclusion: With farmer consent, a veterinarian from one of the eight participating clinics included each case following a farm call reporting a sick cow. In cases where mastitis was suspected, a standardized clinical scoring sheet was completed based on field experience and the literature (Table S1) [15]. This included rectal temperature, rumen fill, ruminal motility, ocular congestion, enophthalmos, skin tent duration, behavior, and degree of depression. Cows were classified as severe mastitis cases when the clinical score was ≥3. A score ≥6 was considered indicative of shock. Animals already under treatment could be included provided that previous treatments were recorded. Antibiotic-treated cows at inclusion were accepted. None of the cows had been vaccinated against mastitis pathogens. The study period covered the year 2025, with a target inclusion of at least 250 cases.

Treatment: Cows with a clinical score ≥3 received a single intravenous injection of marbofloxacin (10 mg/kg) and flunixin (2.2 mg/kg), provided no other NSAID had been administered within the previous 24 h. No intramammary treatment was administered during the first 24 h post-inclusion. When the clinical score was ≥6, cows received 3 L of 7.2% hypertonic saline solution. If spontaneous water intake did not occur thereafter, oral drenching was performed. Calcium administration was left to the discretion of the attending veterinarian but was systematically recorded. Fluid therapy, with or without drenching, was repeated every 12 h until resolution of shock. Subsequent treatments were determined by the attending veterinarian based on bacteriological results and clinical evolution. No additional antibiotic or anti-inflammatory treatment was permitted within the first 24 h after inclusion. After 24 h, additional antimicrobial therapy was restricted to cases involving Gram-positive intramammary infections.

Additional samples: Microbiological procedures across the eight clinics followed guidelines inspired by the National Mastitis Council [41].

Briefly, 30 µL of well-mixed milk were streaked onto three culture media using a sterile calibrated loop:

1. 5% sheep blood agar (COS–bioMérieux, Lyon, France) for sample quality assessment,
2. 5% sheep blood agar supplemented with nalidixic acid (15 mg/L) and colistin sulfate (10 mg/L) (CNA–bioMérieux) for Gram-positive selection,
3. Hektoen enteric agar (HEKT–bioMérieux) for detection of Enterobacteriaceae.

Plates were incubated aerobically at 37 °C and read at 12, 24, and 48 h. Bacterial identification was performed using standard laboratory methods described elsewhere [20].

Catalase testing (3% H_2_O_2_) was performed on colonies grown on CNA. Colonies that were CNA-positive, catalase-positive, and exhibited double hemolysis, or were coagulase-positive, were identified as *Staphylococcus aureus*. Gram staining was performed on other CNA-positive catalase-positive colonies to distinguish non-aureus staphylococci (NAS) from *Bacillus* spp., *Corynebacterium* spp., yeasts, *Prototheca* spp., and fungi.

For Gram-positive catalase-negative colonies, aesculin hydrolysis was assessed. The Lancefield grouping test was performed when no reaction was observed after two hours of incubation. Aesculin-positive isolates were subcultured on bile aesculin agar to differentiate *Streptococcus uberis* from *Enterococcus* spp. *Trueperella pyogenes* was identified based on small, slow-growing colonies on CNA agar with delayed β-hemolysis, catalase negativity, aesculin negativity, pleomorphic Gram-positive rods, and characteristic V-shaped arrangements.

For organisms isolated on HEKT agar, subculture was performed on CPSO agar and in triple sugar iron (TSI) medium (bioMérieux). Colony color, growth pattern, and medium reactions were used for identification. Only *Escherichia coli*, *Klebsiella* spp., and *Pseudomonas aeruginosa* were differentiated from other Gram-negative bacteria to avoid misclassification.

*E. coli* appeared as yellow colonies on a salmon background on HEKT agar, fermented glucose and lactose in TSI medium, and formed pink colonies on CPSO agar. *Klebsiella* spp. showed similar biochemical behavior but produced blue colonies on CPSO agar. *Pseudomonas aeruginosa* appeared as green colonies on HEKT agar, with no glucose or lactose fermentation and no gas production in TSI medium; colonies appeared brown on CPSO agar.

Identification procedures were regularly validated against reference laboratories, with consistently high agreement (κ > 0.90).

Samples yielding two bacterial species were classified as “mixed culture,” whereas samples yielding three or more distinct species (≥1 CFU per species) were considered contaminated. A threshold of ≥1 CFU/10 µL was required for detection of major mastitis pathogens (*Staphylococcus aureus*, *Streptococcus agalactiae*, *Streptococcus dysgalactiae*, *Streptococcus uberis*, coliforms, and *Enterococcus* spp.) [42]. For other pathogens, the threshold was 2 CFU/10 µL.

Most isolates were subjected to antimicrobial susceptibility testing using the disc diffusion method according to EUCAST guidelines [43].

For Enterobacteriaceae, discs included: marbofloxacin, sulfamethoxazole/trimethoprim, nalidixic acid, gentamicin, amoxicillin/clavulanic acid, and cefquinome. For staphylococci, discs included penicillin, cefoxitin, marbofloxacin, erythromycin, and lincomycin. For enterococci, discs included ampicillin, cephalexin, erythromycin, and lincomycin. Streptococci were not tested, as they are considered uniformly susceptible to penicillin in France [44].

Clinical outcomes were recorded for all included cases. Follow-up was performed via two structured telephone interviews conducted 5 and 15 days after inclusion. Farmers were asked standardized questions regarding changes in general condition, milk production, and the status of the affected quarter (Table S2).

Statistical analysis: Descriptive statistics were first performed using R statistical software. The distribution of the variable of interest (score) was observed, and normality was rejected using the Shapiro–Wilk test. The median, range and interquartile range were therefore reported to describe their distribution. The association between this variable and cows’ mortality was therefore tested using a non-parametric Wilcoxon rank sum test. The level of statistical significance was set at *p* < 0.05.

The association between categorical data such as rectal temperature, clinical score, recumbency at inclusion day and mortality were explored using a Chi-squared or Fisher exact test (if one of the cells had a sample size <5). For contingency variables with 3 or more categories (temperature, score), post hoc tests were performed using Bonferroni correction to determine categories associated with different mortality probability.

## 5. Conclusions

The management of severe mastitis remains challenging for practitioners, with substantial economic and clinical losses still frequently observed. The use of fluoroquinolones raises a therapeutic and ethical dilemma. These critically important antimicrobials exhibit prolonged environmental persistence, and their use is therefore subject to strict regulatory restrictions and should be minimized whenever possible. However, severe mastitis is a life-threatening condition, and fluoroquinolones, due to their pharmacological and pharmacokinetic properties, are particularly well suited for its treatment. They demonstrate high efficacy against the main pathogens associated with septic shock, for which rapid bacterial elimination is essential. The use of fluoroquinolones, despite their critically important status and the strict framework governing their use, may prove valuable, particularly in a context where rapid bacteriological diagnosis is available, enabling targeted therapy and limitation of antibiotic use. This study is only observational: it does not constitute proof of the superiority of fluoroquinolones in the treatment of this type of mastitis, which could only be demonstrated by a properly randomized comparative study with a positive or negative control group.

Several strategies may help reduce mortality associated with severe mastitis. Optimization of immune function through balanced and well-supplemented nutrition, particularly with antioxidant support, as well as proper management of dietary transitions, is essential. Immunity may also be enhanced through vaccination strategies, including vaccines based on the J5 *E. coli* mutant, which have been shown to reduce the severity of Gram-negative infections. Early detection remains a critical factor in improving outcomes. In human medicine, even short delays in the management of sepsis have been associated with increased mortality risk, highlighting the importance of prompt intervention in severe systemic infections.

## Figures and Tables

**Table 1:** Distribution of lactation numbers of included cows (79 missing points of data).

	Lactation Number							
	1	2	3	4	5	6	7	>7
Number of cases	23	40	73	42	29	16	12	9
Percentage	9.4	16.4	29.9	17.2	11.9	6.6	4.9	3.7

**Table 2:** Distribution of clinical cases by the stage of lactation at which they occurred (22 missing points of data).

	Time Relative to Calving					
	−48 h to +48 h	48 h to 3 weeks	3 weeks to 2 months	2 months to 5 months	>5 months	Dry off
Number of cases	43	39	45	78	83	13
Percentage	14.30	13	15	25.90	27.60	4.30

**Table 3:** Percentage of clinical cases exhibiting one or more of the clinical features associated with a deterioration in general condition (281 complete clinical records).

Clinical Signs	Prevalence (%)
Impaired ruminal motility	86.8
Depression	73.3
Decreased or absent appetite	70.7
Gait disturbance (other than recumbency)	58.7
Recumbency	26.3
Signs of dehydration	52.0
Ocular congestion	38.0
Hyperthermia (>39.4 °C)	41.6
Mild hyperthermia (39.1 °C–39.4 °C)	17.5
Normothermia (38.0 °C–39.0 °C)	31.9
Hypothermia (<38.0 °C)	9.0

**Table 4:** Distribution of bacteriological results when only one type of pathogen was isolated.

	Isolated Pathogens	Number of Isolates	Percentage
	*Escherichia coli*	176	62.9
Gram-negative bacteria	*Klebsiella* spp.	20	7.1
	*Pseudomonas*	9	3.2
	Other Gram-negative	5	1.8
	*Staphylococcus aureus*	15	5.4
Gram-positive bacteria	*Streptococcus uberis*	19	6.8
	*Streptococcus dysgalactiae*	8	2.9
	*Trueperella pyogenes*	4	1.4
	Other Gram-positive (CNS, *Enterococcus* spp., *Bacillus cereus*)	12	4.3
	yeast	2	0.7
	No growth	10	3.6

**Table 5:** Distribution of bacteriological results when two pathogens were isolated.

Two Pathogens Isolated	Number of Isolates
*Escherichia coli* + *Streptococcus uberis*	18
*Escherichia coli* + *Staphylococcus aureus*	4
*Escherichia coli* + other Gram-positive	8
*Escherichia coli* + *Streptococcus dysgalactiae*	2
*Escherichia coli* + *Klebsiella*	2
*Escherichia coli* + *Trueperella pyogenes*	1
*Klebsiella* + *Streptococcus uberis*	1
*Klebsiella* + other Gram-positive	1
*Staphylococcus aureus* + *Streptococcus dysgalactiae*	2
*Streptococcus uberis* + other Gram-positive	1

**Table 6:** Percentage of susceptibility of 188 strains of *E*. *coli* to commonly used antibiotics for this indication determined using the disc diffusion method.

	Susceptible	Intermediate	Resistant
Amoxycillin + clavulanic acid	35.2	47.8	17
Cefquinome	98.5		1.5
Nalidixic acid	93.7	2.9	3.4
Marbofloxacin	100		
Gentamycin	99.3	0.7	
Sulfamethoxazole + trimethoprim	79.9		20.1

**Table 7:** Statistical comparison of the mean clinical scores between cows that survived and those that died or were euthanized (clinical score missing for 3 cows).

Characteristic	Alive N = 277	Dead N = 43	*p*-Value ^2^
Score	12.1 ^1^ (6.9–17.3)	18.2 ^1^ (13.5–22.9)	<0.001

^1^ = meaning of the displayed value, ^2^ = statistical test used.

**Table 8:** Overview of the total number of cows and the number that died or were euthanized in each of the different clinical score classes (clinical score missing for 3 cows).

Clinical Score Classes	Number of Cases	Dead/Euthanized Cows	Mortality Rate (%)
3 to 5	26	0	0
6 to 9	78	1	1.3
10 to 14	100	7	7
15 to 19	77	21	27.3
20 to 24	29	8	27.6
≥25	10	6	60

**Table 9:** Distribution of pathogens isolated from dead or euthanized cows.

Pathogen(s) Isolated	Number of Dead/Euthanized Cows
*Escherichia coli*	25
*Escherichia coli* + other pathogen	6
*Klebsiella*	2
*Staphylococcus aureus*	2
Other Gram-positive	4
*Streptococcus uberis*	1
*Streptococcus uberis* + other Gram-positive	1
No growth	2

**Table 10:** Assessment of milk production in cows 15 days after they were included (251 valid records; the others had missing data or were beef cattle).

	Maintaining Full Milk Production	Maintaining over 50% of Milk Production	Maintaining Under 50% of Milk Production	Loss of Milk Production
Number of case	56	94	34	67
Percentage	22.3	37.5	13.5	26.7

**Table 11:** Assessment of the condition of the affected quarter of the cows 15 days after they were included (253 valid records; the others had missing data or were beef cattle).

	The Quarter Has Been Fully Cured	The Quarter Is Still Swollen/Hard, but the Milk Is Normal	The Quarter and the Milk Are Still Being Modified	The Quarter Is Dry
Number of cases	112	35	20	86
Percentage	44.3	13.8	7.9	34.0

## Data Availability

The data is organized into two Excel files (one shared with all participating veterinary facilities and another containing all available clinical data) and is available upon request.

## Supplementary Materials

The following supporting information can be downloaded at: https://www.mdpi.com/article/10.3390/antibiotics15060538/s1, Table S1: Shared Excell database; Table S2: Scoring of clinical cases.
