# Supplementary material for: Etiological and Epidemiological Characteristics of Severe Mastitis and the Outcomes Treatment Following a Single Dose of Fluoroquinolones Administered During On-Farm Veterinary Interventions
Source: Antibiotics (Basel). 2026 May 25;15(6):538. doi: 10.3390/antibiotics15060538 (PMC13295865; doi:10.3390/antibiotics15060538)
Supplement: Supplementary file 1 [file antibiotics-15-00538-s001.zip › antibiotics-4236087-supplementary.pdf]

Table S1. Shared Excell database

Parameters	Outcome	rating: Classification	rating: Clinical grade assigned	Score
Rectal temperature (RT)	……°….c	RT > 39.4°c	3	
		39.0°c < RT ≤ 39.4°c	1	
		37.9°c < RT ≤ 39.0°c	0	
		RT ≤ 37.9°c	4	
Heart rate		……………………bpm		
Respiratory rate		……………………bpm		
Ruminal fill score	……	Score 3, 4 or 5	0	
		Score 2	2	
		Score 1	4	
Ruminal motility (by 2 mn)	………/2 mn	3 – 4 contractions	0	
		1 – 2 contractions	2	
		O contraction	4	
Ocular mucous membranes		Normal	0	
		Moderate congestion (1 single scleral visible vessel)	2	
		Severe congestion (>1 scleral visible vessel)	4	
Enophtalmia		Absent	0	
		Moderate (< 2mm)	2	
		Severe (> 2mm)	4	
Persistence of the skin fold at the neckline		< 2 seconds	0	
		2 – 3 seconds	1	
		3 – 6 seconds	2	
		> 6 seconds	4	
Ability to move	Normal locomotion, quick rising		0	
	Reluctant to move, slow to respond		2	
	Staggering, difficult rising		4	
	Sternal recumbency		6	
	Lateral recumbency		8	
Depression	Normal behavior, attentive cow		0	
	Noticeable change in behavior, sad cow		2	
	Marked behavioral change, seems detached from its surroundings, ears down		4	
**Clinical Score**				

Table S2. Scoring of clinical cases

Case number : Call's date :

☐ 1st call (D+5) ☐ 2nd call (D+15)

Herd :

Ear tag of the cow :

A Assessment of the cow's general condition

- ☐ 0 : the cow is cured
- ☐ 1 : the cow is feeling better
- ☐ 2 : the cow is still very weak
- ☐ 3 : the cow is dead or has been euthanized

B Assessment of milk production

- ☐ 0 : Maintaining full milk production
- ☐ 1 : Maintaining over 50% of milk production.
- ☐ 2 : Maintaining under 50% of milk production.
- ☐ 3 : Loss of milk production

C Assessment of the quarter

- ☐ 0 : The quarter has been fully cured
- ☐ 1 : The quarter is still hard, but the milk is normal
- ☐ 2 : The quarter and the milk are still being modified
- ☐ 3 : The quarter is dry
